# Biomechanical testing of transcapsular meniscal repair

**DOI:** 10.1186/s40634-017-0075-7

**Published:** 2017-01-25

**Authors:** Ryo Iuchi, Tatsuo Mae, Konsei Shino, Tomohiko Matsuo, Hideki Yoshikawa, Ken Nakata

**Affiliations:** 1 Sports Orthop. Center, Yukioka Hospital, Osaka, Japan; 2 Department of Orthopaedic Surgery, Osaka University Graduate School of Medicine, Osaka, Japan

## Abstract

**Background:**

All of previous biomechanical studies on meniscal repair have examined the meniscus itself without synovial membrane and capsule, although in the clinical setting, the meniscal repair is generally performed including capsule. Therefore, biomechanical properties of transcapsular meniscal repair are unclear. Thus, this study aimed to clarify the biomechanical properties of transcapsular meniscal repair.

**Methods:**

In 70 porcine femur–medial meniscus–tibia complexes with capsules, longitudinal meniscal tears were repaired using different suture techniques (inside-out or all-inside technique), suture methods (vertical or horizontal methods), and numbers of sutures (single or double). A cyclic loading test between 5 and 20 N for 300 cycles was performed followed by a load-to-failure test.

**Results:**

Tears repaired by the all-inside technique presented significantly larger widening (0.88 ± 0.38 mm) than those by the inside-out technique (0.51 ± 0.39 mm) during the cyclic loading test (*P* = 0.035). The horizontal suture presented significantly lower ultimate failure load (62.5 ± 15.5 N) in the all-inside technique than in the vertical suture (79.7 ± 13.0 N; *P* = 0.018). The stacked suture had a significantly higher failure load (104.6 ± 12.5 N) than the parallel suture (83.3 ± 12.6 N; *P* = 0.001). Furthermore, the double suture presented significantly higher failure loads (83.3 ± 12.6 N and 104.6 ± 20.4 N) than the single suture with both inside-out (58.8 ± 8.3 N; *P* = 0.001) and all-inside (79.7 ± 13.0 N; *P* = 0.022) techniques.

**Conclusions:**

Upon comparison of the suture techniques, the inside-out technique provided a more stable fixation at the repair site than the all-inside technique during the cyclic test. Among the suture methods, the vertical suture had more desirable biomechanical properties than the horizontal suture as demonstrated by smaller widening during the cyclic test and the larger load to failure. The stacked suture created a stronger fixation than the parallel suture. In terms of the number of sutures, the double suture had superior biomechanical properties compared with the single suture.

## Background

Meniscal repair is one of the meniscal treatment alternatives widely performed to restore the functions of injured menisci (Cannon and Morgan 1994; DeHaven 1985; Henning et al. 1998; Horibe et al. 1996; Morgan 1991; Rubman et al. 1998; Tachibana et al. 2010; Warren 1985). A successful repair requires stabilization of the torn meniscal tissue during the healing process. Many factors, including repair techniques, repair methods, and the number of sutures, may influence the stabilization of the repair site (Barber and Herbert 2000; Chang et al. 2005; Horibe et al. 1995; Kocabey et al. 2006; Post et al. 1997; Rimmer et al. 1995).

Although there are several available meniscal repair techniques, such as inside-out, outside-in, and all-inside techniques (Barber et al. 2009; Cannon and Morgan 1994; DeHaven 1985; Henning et al. 1998; Morgan 1991), the inside-out technique is reported to be the gold standard (Henning et al. 1998). Horibe et al. reported that 97 (73%) of 132 menisci repaired using the inside-out technique had completely healed by second-look arthroscopic evaluation (Horibe et al. 1995). Noyes et al. demonstrated that a successful meniscal repair with apparent retained function was achieved and maintained during 10–20 years postoperatively in 62% patients using an inside-out multiple vertical divergent suture technique (Noyes and Barber-Westin 2002). However, inside-out suture techniques required an additional incision to reduce the risk of neurovascular injuries and an additional surgeon to perform the sutures (Morgan 1991; Warren 1985). All-inside meniscal repair devices, first reported in 1993, are widely used today (Albrecht-Olsen et al. 2999; Barber et al. 2004, 2012). Barber et al. described that the all-inside repair devices were similar to conventional inside-out repairs in the load to failure tests using porcine menisci (Barber et al. 2009). Zantop et al. also reported no significant difference between the all-inside repair and conventional inside-out repair in cyclic loading tests using human menisci (Zantop et al. 2005). Therefore, flexible all-inside and inside-out techniques provided similar stability. To achieve a successful meniscal repair, moreover, suture methods, such as vertical and horizontal sutures, and the number of sutures are quite critical (Cannon and Morgan 1994; Henning et al. 1998). Kohn and Siebert recommended the vertical suture because the load at failure of vertical suture was superior to that provided by horizontal suture in a cadaver meniscus simplex model (Kohn and Siebert 1989).

However, none of the previous biomechanical studies has truly represented the clinical situation. They have examined the meniscus itself without synovial membrane and capsule, (Barber et al. 2004, 2009, 2012; Barber and Herbert 2000; Becker et al. 2002; Chang et al. 2005; Kocabey et al. 2006; Kohn and Siebert 1989; Post et al. 1997; Rimmer et al. 1995; Rosso et al. 2011; Seil et al. 2000; Song and Lee 1999; Zantop et al. 2005). although in the real clinical setting, the meniscal repair is generally performed by tying knots or putting anchors on capsules. Therefore, biomechanical properties of transcapsular meniscal repair are unclear. Thus, this study aimed to clarify the differences in biomechanical characteristics between the all-inside and the inside-out techniques, the difference of biomechanical characteristics between two suture methods; the use of either vertical or horizontal, parallel or stacked suture in the double sutures; and the effect of numbers of sutures on the biomechanical strength in a transcapsular suture model using the femur-meniscus-tibia complex with capsule. The hypotheses of the study were that 1) the biomechanical characteristics of the all-inside technique would be similar to those of the inside-out technique, 2) the vertical suture would provide superior biomechanical characteristics compared with horizontal suture, and 3) multiple sutures would provide superior biomechanical characteristics compared to single sutures.

## Methods

### Specimens and preparations

**Fig. 1:**
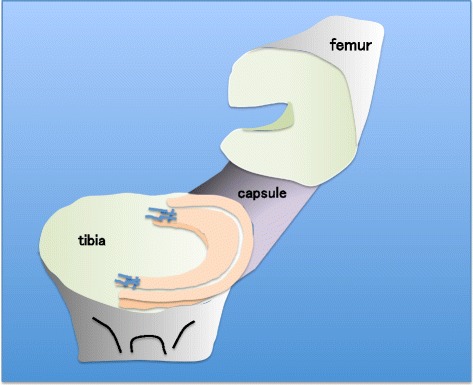
The femur - medial meniscus - tibia complex and preparation for meniscal tear. Medial meniscus was sharply cut off in 3-mm width from the capsule. No.2 braided polyester sutures were whip-stitched at the both horns

Seventy porcine knees, each of 6 months of age were used. All specimens were kept frozen at −20 °C and then allowed to thaw at 4 °C for 24 h. In order to prepare the femur-medial meniscus-tibia complex with a synovial membrane and capsule, the patellae, patellar tendon, muscles, cruciate ligaments, lateral collateral ligament, lateral meniscus, and lateral half of the joint capsule were removed. Then, the medial meniscus was sharply cut off with the scalpel, leaving a peripheral meniscus at a distance of 3 mm from the capsule (Kohn and Siebert 1989; Zantop et al. 2005). No. 2 braided polyester sutures were whip-stitched at the anterior and posterior horns on the cut-off meniscus (Fig. 1).

### Meniscal repairs

**Fig. 2:**
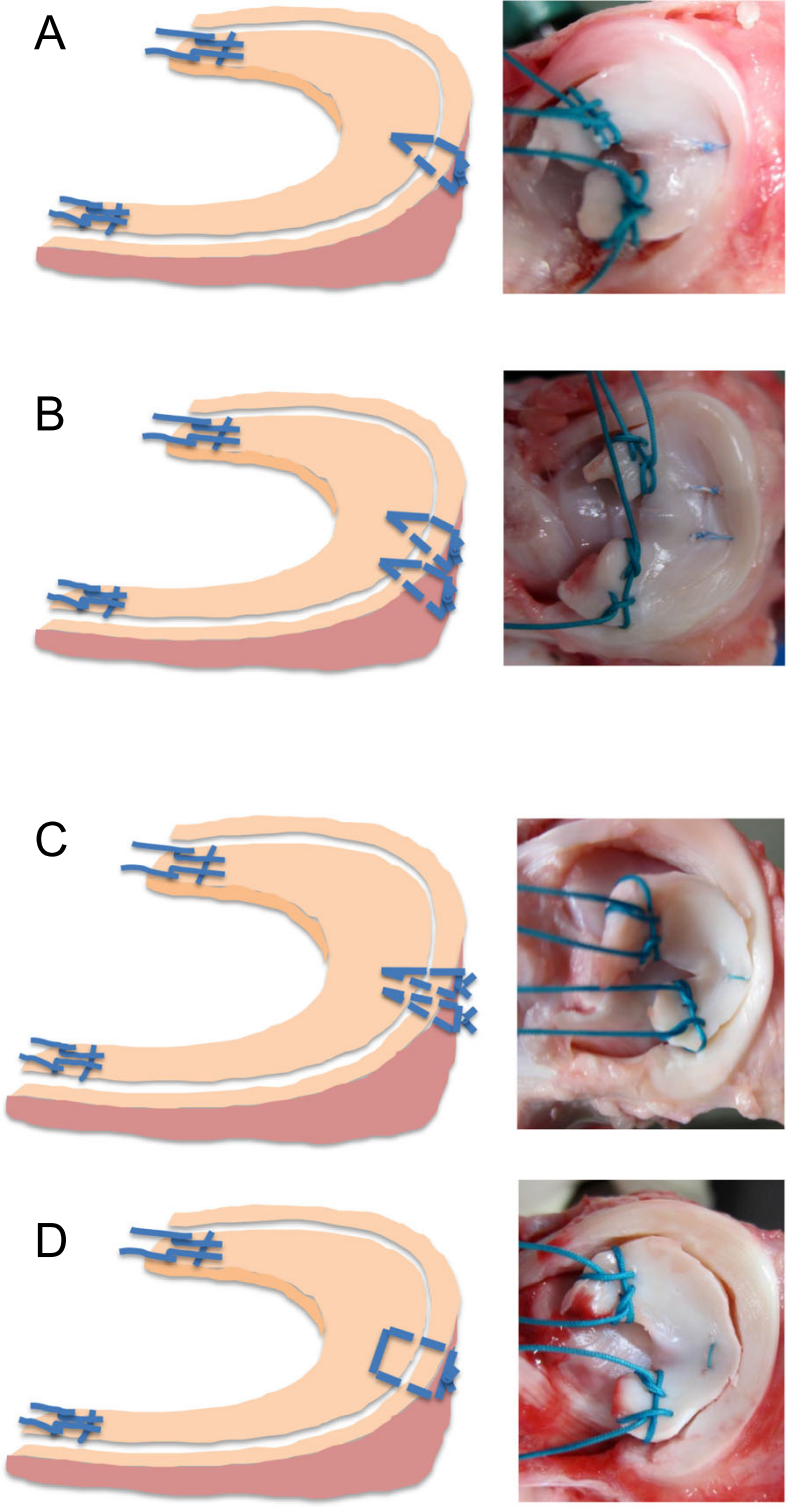
Suture methods. **a** Single vertical suture. **b** Double vertical suture. Repairs were performed with vertical suture at intervals of 5-mm. **c** Stacked vertical suture. The superior sutures were placed first close to the superior gap, and the inferior sutures were then placed. In the photo, though only one suture was on the upper side, another suture was on the lower side. **d** Horizontal suture. The distance between first and second delivery needle were 5-mm

The 70 specimens were divided into seven groups, while the menisci were repaired using the inside-out (Group 1–4) or all-inside (Group 5–7) techniques (Fig. 2, Table 1).

**Table 1:** Repair group

Group	Technique	Device	Number	Method
1	inside-out	No. 2-0 braided polyester suture	1	vertical
2	inside-out	No. 2-0 braided polyester suture	2	parallel
3	inside-out	No. 2-0 braided polyester suture	2	stacked
4	inside-out	No. 2-0 braided polyester suture	1	horizontal
5	all-inside	Ultra FastFix	1	vertical
6	all-inside	Ultra FastFix	2	parallel
7	all-inside	Ultra FastFix	1	horizontal

Meniscal lesions were repaired using the following 2 devices. For the inside-out technique, a No. 2-0 braided polyester suture (Stryker, Japan) was used and tied to the capsule by hand with four square knots. For the all-inside technique, the Ultra FasT-Fix (Smith & Nephew Endoscopy, Andover, MA, USA) composed of two 5-mm PEEK anchors and No. 0 Ultrabraid suture was used in accordance with the manufacturer’s instructions.

The needles/anchors for meniscus were inserted within a 3-mm distance from the outer margin of the detached meniscus. The other needles/anchors in the vertical suture were inserted into the remaining meniscus connected to the capsule, while those in the horizontal suture were inserted 3 mm apart from the outer margin of the detached meniscus and 5 mm apart from each other. Then, all sutures tying in the inside-out technique was performed on the capsule manually by one surgeon having 10 years of experience in arthroscopic surgery.

### Tensile testing

Biomechanical testing was conducted on a material testing machine AUTOGRAPH AG-IS (SHIMADZU, Kyoto, Japan). Femur and tibia were settled into custom-made holders, and No. 2 polyester sutures were stitched to both horns of the medial meniscus and were securely fastened to the clamp connected to the load cell. This set-up allowed a consistent application of force to the repair site.

**Fig. 3:**
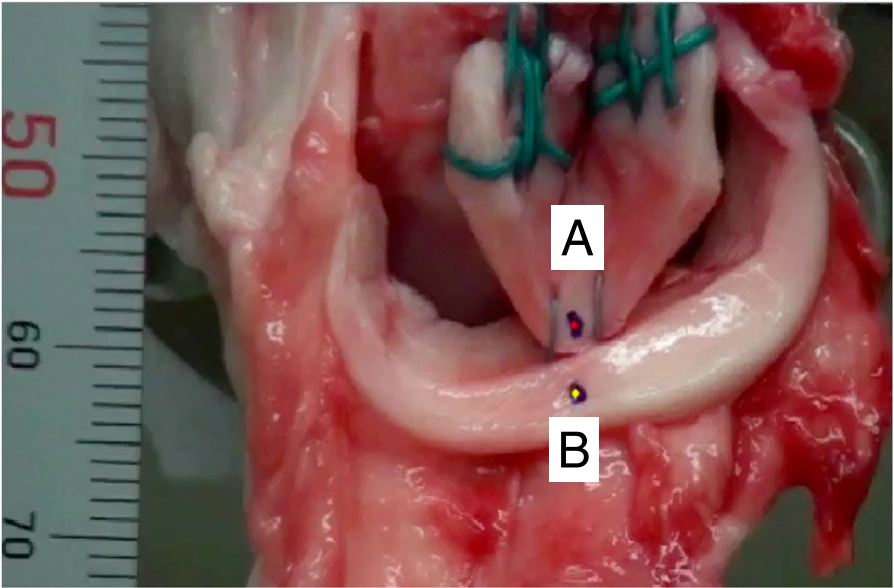
Video analysis. The distance between two dots (**a**, **b**) on the both sides of repaired meniscus was measured as the gap using the image analysis software in the inside-out technique with double vertical suture

After marking two small dots on both sides of the repaired meniscus, a cyclic loading test was performed between 5 and 20 N at a rate of 200 mm/min for 300 cycles. Following the cyclic loading test, a load to failure test was performed at a rate of 5 mm/min (Barber et al. 2009; Chang et al. 2005; Kohn and Siebert 1989). These procedures were recorded by a video recorder (HDR-CX370V: SONY, Tokyo, Japan), whereas the distance between the two previously-marked dots was measured using an image analysis software (DIPP-Motion Pro2D: DITECT, Tokyo, Japan) (Fig. 3). This system can measure the distance by at least one tenth of one millimeter.

By analyzing the distance between two dots, the widening at the repaired site was measured more precisely, as the clamp measurement includes the slippage at each clamp and stress-relaxation of the soft tissue around the knee (Rosso et al. 2011).

Then the widening of suture after cyclic loading, the ultimate failure load, the stiffness during load-to-failure test, and the mode of failure were documented. The widening of repaired site after cyclic loading was defined as the difference between the distance of two dots at the first cycle and that at the last cycle under a load of 5 N. The stiffness was calculated from the linear region of the load-displacement curve.

For those data, we assessed the following three subjects:

1. Suture techniques:
  Inside-out versus all-inside techniques
  - Group 1 vs. 5 in the vertical suture
  - Group 4 vs. 7 in the horizontal suture
2. Suture methods:
  (A) Vertical versus horizontal sutures in the same suture technique
    c) Group 1 vs. 4 in the inside-out technique
    d) Group 5 vs. 7 in the all-inside technique
  (B) Parallel versus stacked suture in the double sutures
  e) Group 2 vs. 3 in the inside-out technique
3. Number of sutures:

f) Group 1 vs. 2 in the inside-out technique
g) Group 5 vs. 6 in the all-inside technique

### Statistical analysis

The study compared data for each group using a 1-way analysis of variance (ANOVA). Thus, the Mann-Whitney’s U test was used to detect the significance in differences in suture techniques, suture methods, and the number of sutures with a statistical analysis software (PASW Statistics 18.0: SPSS Chicago, IL, USA). Statistical significance was set at *P* < 0.05.

## Results

There were no significant differences among groups using a 1-way ANOVA.

The widening of the suture after cyclic loading was the smallest in group2, while the largest ultimate failure load was found in groups 3 and 6 (Table 2).

**Table 2:** Widening of suture after cyclic load, ultimate failure load and stiffness

Group	Widening after cyclic load (mm)	Ultimate failure load (N)	Stiffness (N/mm)
1	0.51 ± 0.39	58.8 ± 8.3	35.4 ± 14.8
2	0.38 ± 0.17	83.3 ± 12.6	45.9 ± 19.9
3	0.41 ± 0.15	104.6 ± 12.5	79.0 ± 48.0
4	1.21 ± 0.59	62.4 ± 5.0	43.6 ± 26.0
5	0.88 ± 0.38	79.7 ± 13.0	44.3 ± 19.2
6	0.59 ± 0.36	104.6 ± 20.4	92.3 ± 76.2
7	1.23 ± 0.74	62.5 ± 15.5	45.1 ± 24.5

All of the sutures failed by suture breakage, except for two sutures in the all-inside technique (Table 3).

**Table 3:** Modes of failure

Group	Suture breakage	Anchor pulling out
1	10	
2	10	
3	10	
4	10	
5^a^	8	1
6	10	
7	9	1

^a^ One in Group 5 failed the anchor fixation by pulling out during the cyclic loading test

### Suture techniques

Repair portion with the all-inside technique presented significantly larger widening than those with the inside-out technique during cyclic loading test using vertical suture (*P* = 0.035). Conversely, in the load to failure test, sutured meniscus with the all-inside technique presented a significantly higher load than those sutures with the inside-out technique using vertical suture (*P* = 0.001) (Table 4).

**Table 4:** Findings of statistical analysis

The subject of comparison	Widening after cyclic load	Ultimate failure load	Stiffness
①Suture techniques			
Group 1 vs. Group 5	*P = 0.035	*P = 0.001	*P* = 0.442
Group 4 vs. Group 7	*P* = 0.734	*P* = 0.921	*P* = 0.921
②Suture methods			
(A) vertical vs. horizontal suture			
Group 1 vs. Group 4	*P = 0.002	*P* = 0.247	*P* = 0.762
Group 5 vs. Group 7	*P* = 0.278	*P = 0.018	*P* = 0.829
(B) parallel vs. stacked suture			
Group 2 vs. Group 3	*P* = 0.622	*P = 0.002	*P* = 0.131
③Number of sutures			
Group 1 vs. Group 2	*P* = 0.762	*P = 0.001	*P* = 0.462
Group 5 vs. Group 6	*P* = 0.09	*P = 0.022	*P = 0.036

*There was statistically the significant difference between two groups. (*p* < .05)

### Suture methods

#### Vertical versus horizontal sutures

Horizontal sutures presented significantly larger widening during cyclic loading using the inside-out technique (*P* = 0.002). The horizontal suture showed significantly lower ultimate failure load than the vertical suture in the all-inside technique (*P* = 0.018) (Table 4).

#### Parallel versus stacked suture in the double sutures

The stacked suture had a significantly higher failure load than the parallel suture (*P* = 0.002).

### Numbers of sutures

When using double sutures, there was a significantly higher failure load than with single sutures with both the inside-out and all-inside techniques (*P* = 0.001; *P* = 0.022). In the all-inside technique, the double suture showed significantly higher stiffness than the single suture (*P* = 0.036) (Table 4).

## Discussion

The principal findings of this study were that the inside-out technique provided more stable fixation than the all-inside technique during cyclic loading, the vertical suture had better biomechanical characteristics than the horizontal suture, and the double sutures showed higher load to failure than the single sutures. To our knowledge, this is the first study to clarify the biomechanical properties of the transcapsular meniscus suture using the femur-medial meniscus-tibia complex. These data may be more clinically useful than those previously obtained because our study model represented the actual clinical situation quite closely, as compared to the previous study models without capsule.

The inside-out technique is considered the standard for meniscal repair; however, it requires an additional skin incision (Cannon and Morgan 1994; Henning et al. 1998). The all-inside technique has the advantage of not requiring an additional incision, leading to a decrease in operative time (Morgan 1991; Warren 1985). In the case of repairing with the vertical suture generally used for a longitudinal tear, Barber et al. reported that there was no significant difference in the displacement during cyclic test between all-inside (MaxFire and Ultra FasT-Fix) and inside-out techniques (No. 2-0 Mersilene) in porcine menisci (Barber et al. 2009). Rosso et al. also reported that there was no significant difference in the displacement during the cyclic test between the all-inside technique (Ultra FasT-Fix) and its matched inside-out technique (No. 0 Ultrabraid suture) in porcine menisci themselves (Rosso et al. 2011). However, in this study, while mimicking the clinical transcapsular repair, the widening of the suture was significantly smaller after the cyclic loading test in the inside-out technique than that in the all-inside technique using the vertical suture. Then, the lubrication of the capsule might have had an influence on the results. Therefore, when performing a meniscal repair using the all-inside technique with the vertical suture, surgeons might need to knot the suture more tightly in anticipation of this potential slack.

Either the vertical or horizontal suture method is conventionally used in meniscal repair (Henning et al. 1998; Post et al. 1997; Song and Lee 1999). This study showed that the vertical suture method was superior in biomechanical properties to the horizontal suture method. Rimmer et al. also reported that failure load (67 N) of vertical sutures was superior to that of horizontal sutures (29.3 N) when performed on cadaveric menisci using the inside-out technique with No. 3-0 Ethibond suture (Rimmer et al. 1995). Becker et al. investigated the displacement of both suture methods after cyclic loading in a cadaver model, and reported that vertical sutures provided significantly less displacement in comparison with the horizontal sutures (Becker et al. 2002). The horizontal sutures encircled parallel to the circumferential meniscal fibers; whereas, the vertical suture encircled perpendicular to those fibers (Post et al. 1997; Rimmer et al. 1995). Therefore, higher rate of partial tissue failures could occur in the horizontal suture and result in lower stability during the cyclic test (Seil et al. 2000). Moreover, it was difficult to equally load to both sutures during tests in horizontal suture technique. Then, one suture in the horizontal suture might receive higher load with earlier rupture because of the distance between the two sutures. This imbalance of loading can exist in clinical situation as well as in the experimental setting. Thus, further caution is needed for period of weight bearing after meniscal horizontal suture.

Concerning the double sutures in the inside-out technique, the parallel sutures had a significantly lower failure load than the stacked sutures. As previously described, the imbalance of loading to sutures must occur in this comparison because it is difficult to simultaneously add the same load to both sutures. Thus, the stacked suture could be expected to provide a more stable fixation in the clinical setting.

In a clinical setting, the meniscal repair is generally performed for an unstable and large tear using multiple sutures. Henning recommended that three to four sutures should be placed in the posterior third of the medial meniscus (Henning et al. 1998). However, there have been no biomechanical reports on the effect of multiple sutures. In this study, there were no significant differences of widening after cyclic loading with either technique. Thus, both single and double suture could stabilize the meniscal tear with small stress under this cyclic loading condition. On the other hand, as the double sutures presented significantly higher ultimate failure load and stiffness than the single suture, double sutures might be more suitable for the stabilizing meniscal tear under a weight bearing condition. Therefore, we consider that multiple sutures are desired when the meniscal repair is required for an unstable large tear.

### Limitations

There were some limitations in this study. First, the porcine meniscus was used instead of a human meniscus. Most of the available cadavers were elderly patients who were likely to have degenerative menisci, suggesting that the biomechanical results vary if menisci of poor quality were used. We selected the porcine menisci because we could achieve results with a higher precision level with no damages. Moreover, several studies revealed that the porcine menisci had properties comparable to that of human menisci (Barber et al. 2009; Joshi et al. 1995). Secondary, the suture number was not proportional to the tear length in this model and three or more sutures should also have been investigated. The large tear such as this model usually requires many sutures. However, in case of repairing with multiple sutures adapted for the large tear, there is a concern that the even stress could not be loaded to each suture during load-to-failure test. Thus, in this study, single or double suture were used for the large tear just to clarify the difference among the suture techniques or methods. Third limitation of this study is that the widening of the suture was measured at the only upper side of the meniscus. Calipers or actuator measurements were generally used to measure the widening of the suture in most previous reports, though calipers can also measure the only upper side. Moreover, the actuator measurements have the possibility of the deformation of meniscus and the slippage in the holder or the clamp on the testing machine. Therefore, it is considered that our measurement with the video recorder and the image analysis software can measure the widening of the suture more accurately despite of this limitation. Fourth limitation is that we tested the worst-case scenario, whereby the load is applied parallel to the axis of the repair suture. Although the exact forces across a meniscus repair in vivo are unknown, the in vivo forces might be more complex than in a unidirectional test setup (Becker et al. 2006; Richards et al. 2008). Despite this limitation, the data obtained in this study can contribute to the improvement of the surgical outcome.

## Conclusion

Upon comparison of the suture techniques, the inside-out technique provided a more stable fixation at the repair site than the all-inside technique during the cyclic test. Among the suture methods, the vertical suture had more desirable biomechanical properties than the horizontal suture as demonstrated by smaller widening during the cyclic test and the larger load to failure. The stacked suture created a stronger fixation than the parallel suture. In terms of the number of sutures, the double suture had superior biomechanical properties compared with the single suture.
